# A Conditional Knockout Mouse Model Reveals a Critical Role of PKD1 in Osteoblast Differentiation and Bone Development

**DOI:** 10.1038/srep40505

**Published:** 2017-01-13

**Authors:** Shao Li, Wanfu Xu, Zhe Xing, Jiabi Qian, Liping Chen, Ruonan Gu, Wenjing Guo, Xiaoju Lai, Wanlu Zhao, Songyu Li, Yaodong Wang, Q. Jane Wang, Fan Deng

**Affiliations:** 1 Department of Cell Biology, School of Basic Medical Sciences, Southern Medical University, Guangzhou 510515, P. R. China; 2 Department of Pharmacology and Chemical Biology, University of Pittsburgh School of Medicine, Pittsburgh, PA 15261, USA

## Abstract

The protein kinase D family of serine/threonine kinases, particularly PKD1, has been implicated in the regulation of a complex array of fundamental biological processes. However, its function and mechanism underlying PKD1-mediated the bone development and osteoblast differentiation are not fully understood. Here we demonstrate that loss of PKD1 function led to impaired bone development and osteoblast differentiation through STAT3 and p38 MAPK signaling using *in vitro* and *in vivo* bone-specific conditional PKD1-knockout (PKD1-KO) mice models. These mice developed markedly craniofacial dysplasia, scapula dysplasia, long bone length shortage and body weight decrease compared with wild-type littermates. Moreover, deletion of PKD1 *in vivo* reduced trabecular development and activity of osteoblast development, confirmed by Micro-CT and histological staining as well as expression of osteoblastic marker (OPN, Runx2 and OSX). Mechanistically, loss of PKD1 mediated the downregulation of osteoblast markers and impaired osteoblast differentiation through STAT3 and p38 MAPK signaling pathways. Taken together, these results demonstrated that PKD1 contributes to the osteoblast differentiation and bone development via elevation of osteoblast markers through activation of STAT3 and p38 MAPK signaling pathways.

Skeletal integrity requires a delicate balance between bone-forming osteoblasts and bone-resorbing osteoclasts. The imbalance between bone formation and bone resorption results in metabolic bone diseases such as osteoporosis. The rate of genesis as well as death of these two cell types is vital for the maintenance of bone homeostasis^1^,^2^.

As the major bone formation cells, osteoblasts differentiate and produce bone matrix during skeletal development^3^. The osteoblast differentiation is often divided into stages of mesenchymal progenitors, preosteoblasts and osteoblast^4^, while the bone formation occurs through two distinct developmental processes: intramembranous ossification and endochondral ossification^5^,^6^. Osteoblast differentiation is controlled by various transcription factors, such as runt-related transcription factor-2 (Runx2) and osterix (Osx), which have been identified as osteoblast lineage controllers^7^. Runx2 plus its companion subunit core binding factor beta (Cbfb) are required for an early step in osteoblast development, whereas Osx is required for a subsequent step, namely the differentiation of preosteoblasts into fully functional osteoblasts^8^. Although osteoblast differentiation and bone development are attributed to bone morphogenetic protein (BMP), fibroblast growth factor (FGF), Wnt and JAK/STAT signaling pathways^4^,^5^,^9^, the molecular mechanism underlying osteoblast differentiation and bone development remains still poorly understood.

The protein kinase D (PKD) family of serine/threonine kinases belongs to the Ca^2+^/calmodulin-dependent protein kinase (CaMK) superfamily. There are three isoforms (PKD1, 2 and 3) of PKD, which are widely distributed in a variety of tissues and exhibit high sequence homology^10^,^11^. Several conserved structure domains are present in PKD, including a diacylglycerol-binding C1 domain and a PH domain that exerts an autoinhibitory function to the kinase activity. PKD can be activated by PKC-mediated trans-phosphorylation of two conserved serine residues (Serine 738/742 in human PKD1) in the activation loop of PKD^12^. Sustained PKD activation can be maintained via PKC-independent autophosphorylation events^13^. PKD plays an important role in propagating signals from G protein-coupled receptors (GPCRs) and growth factor receptors at the cell surface by means of the DAG/PKC/PKD axis.

Current studies show that PKD signaling has been implicated in bone biology. Protein kinase C-independent activation of PKD is stimulated by bone morphogenetic protein-2 (BMP-2) and Insulin-like growth factor-I (IGF-I) in mouse osteoblastic MC3T3 cells^14^. Meanwhile, in human bone marrow progenitor cells (mesenchymal stem cells), the increase of Osx a major osteoblastic transcription factor, is also induced by PKD signaling passway^15^. Moreover, PKD activation contributes to the synergistic induction of osteoblast differentiation and mineralized nodule formation via BMP-7 and IGF-I^16^. In addition, activation of PKD1 induced by BMP2 regulates histone deacetylase 7 (HDAC7) nuclear export, thereby alleviating repression of Runx2-mediated transcription, indicating that PKD-dependent factors beyond attenuation of HDAC7-repressive activity are required for osteoblast differentiation^17^. These studies have implicated PKD signaling in osteoblast function as a mediator of hormonal signaling *in vitro* at the cellular level.

Although attenuated PKD1 kinase activity in heterozygous animals (prkd1+/− mice) showed bone mass and osteoblast function abnormality during pubertal growth^18^, the specific function and mechanism of PKD1 in osteoblasts differentiation and bone development are still not well understood. In this study, we used genetic approaches to create an osteoblast-specific *prkd1*-dificient mouse model in which the *Prkd1* gene flanking exons 12 through 14 were specifically ablated in osteoblasts (*prkd1*^−/−^) by Cre-loxp recombination^19^,^20^. We demonstrated an essential role of PKD1 in bone development and the function of PKD1 in regulating osteoblast activities in mice.

## Result

### Bone-specific deletion of PKD1 in Osx::PKD1^fl/fl^ mice resulted in postnatal growth bone defects

**Figure 1:**
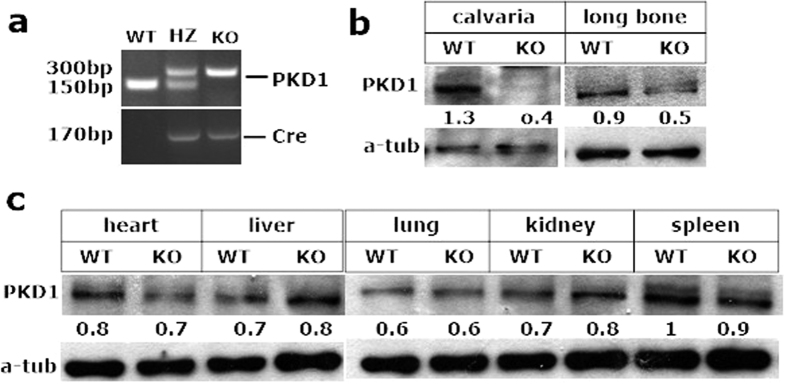
PKD1 was deleted specifically in transgenic mice. (**a**) Representative genotyping PCR for PKD1 floxed and cre transgene relative to wild type PKD1. The genome of littermates knockout (KO) carried two copy of PKD1floxed and one copy of the OSX-Cre gene (Osx::PKD1^fl/fl^ mice), the genome of littermates heterozygou (HZ) carried one copy of PKD1 floxed and the OSX-Cre gene (Osx-Cre::PKD1^fl/+^), and the genome of cre-null littermates control only carried PKD1floxed gene (wild type control, WT). (**b**) Representative Western blotting for the expression of PKD1 and internal control of alpha tubulin (α -tub) in calvariae and long bone derived from 4-week-old KO or WT mice. Uncropped western blot images corresponding to Fig. 1(b,c) were shown in supplementary information file. Tissues were pooled from WT or KO mice (n = 3); experiments were repeated twice using mice from different litters. (**c**) Western blotting for the expression of PKD1 and internal control ofα-tub in heart, liver, kidney, lung and spleen from the same age WT or KO mice. All Band densities of PKD1were normalized to α-tublin content within each sample.

Because PKD1 is commonly expressed in mouse tissues, homozygous deficiency of PKD1 activity in mice is lethal^21^, we used the Cre-loxP recombination system to generate a conditional *prkd1*allele that could be specifically deleted in mouse bone. LoxP sites were inserted into the *prkd1* locus to flank exons 12 through 14, which encoded part of the catalytic domain of PKD1, including the ATP binding motif that was essential for kinase function^19^. As shown in Fig. 1a, deletion of the genomic region of *prkd1* between the loxP sites in a bone-specific manner was confirmed by PCR of mouse genomic DNA, which distinguished WT (150 bp) from heterozygous Osx::PKD1^fl/fl^ (150 and 300 bp) and knockout Osx::PKD1^fl/fl^ (300 bp and 170 bp) mice. In comparison of wild type mice, the expression of PKD1 in Osx::PKD1^fl/fl^ mice was significantly decreased in the calvaria and long bone (Fig. 1b), and weak or unchanged in other tissues (Fig. 1c). These results showed that the bone-specific deletion of PKD1 existed in Osx::PKD1^fl/fl^ mice.

**Figure 2:**
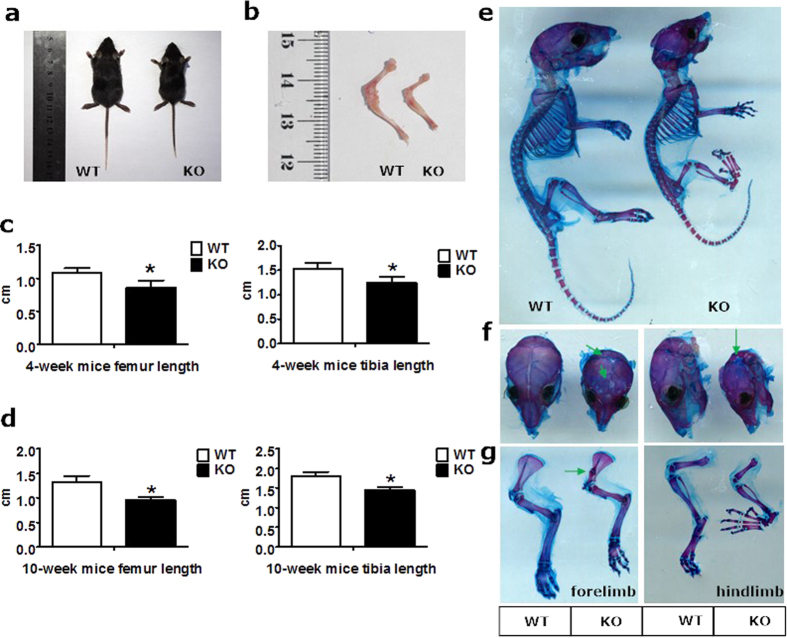
Conditional knockout PKD1 *in vivo* with Osx::PKD1^fl/fl^ mice caused postnatal growth defects. (**a**) Photographic appearance of the 4-week-old WT and KO mice. KO mice were smaller than control mice at 4 weeks. (**b**) Picture of hind limbs from the 4-week-old WT and KO mice. (**c**,**d**) The length changes of femur and tibia from hind limb of 4-week-old and 10 weeks of WT or KO mice. Measurements were presented as mean ± SD (n ≥ 7). The data in each group was analyzed using unpaired, two-tailed Student’s t-test. The level of significance was set at *p < 0.05. (**e**) Whole-mount skeletal was stained by Alizarin red and Alcian blue staining of 14-day-old of WT or KO mice. (**f**) Alcian blue/Alizarin red staining of skulls from WT and KO mice (green arrows showed undeveloped skull). (**g**) Alcian blue/Alizarin red staining of lone bone from WT and KO mice. The arrow reviewed abnormal nodules in scapula.

To evaluate the effect of PKD1 deficiency on the postnatal growth of mouse skeleton, we compared Osx::PKD1^fl/fl^ mice with their sex-matched littermate controls. As shown in Fig. 2a, serious delayed postnatal growth was observed in KO mice. Meanwhile, the body weight of Osx::PKD1^fl/fl^ mice was significantly lower in the 4-week-old or the 10-week PKD1-deficient mice in both sexes than that of littermate control (see Supplementary Fig. 1S). Moreover, the length of femur and tibia was also inhibited in PKD1-deficient mice at the 4-week or 10 week compared with their littermate controls (*p* < 0.05, Fig. 2b,c,d).

To further determine the postnatal skeletal changes during bone development, we examined bone architecture of 14-day (postnatal day 14) mice with the sequential Alcian blue and Alizarin red staining. Compared with littermate controls, the smaller size of bone architecture was observed in Osx::PKD1^fl/fl^ (KO) mice (Fig. 2e). Furthermore, the calvaria bone showed poorer development manifested with a loose mineralized bone structure, delayed suture closure and calvarial defects in 14-day KO mice (Fig. 2f). The length of tibiae and femur in KO mice was shorter than that of control mice with examination of the skeleton of the long bones (Fig. 2g). Similarly, abnormal nodule in the forelimb of scapula in KO mice showed the defects in cartilage ossification (Fig. 2g). In addition, abnormal nodule in scapula from KO mice showed the defects in endochondral ossification (Fig. 2g, left).

### Deletion of PKD1 in Osx::PKD1^fl/fl^ mice led to the bone mass decrease *in vivo*

**Figure 3:**
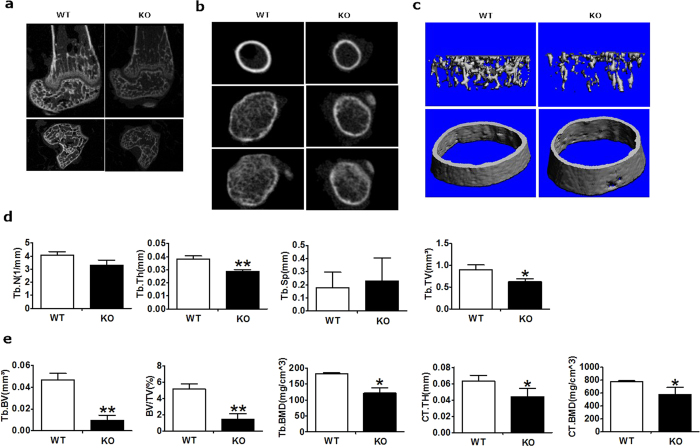
Bone mass was reduced in the distal femur of PKD1 KO mice by micro-CT analysis. (**a**) Less difference of bone mass between WT and KO mice by representative micro-CT images of the distal femur (upper panel) and the structure of metaphyseal (lower panel) trabecular bone. (**b**) Micro-CT images of cortical bone in the mid-diaphysis (upper panel) and trabecular bone in the metaphysis (middle and lower panel). (**c**) Three-dimensional models images of micro-CT analyses of the structure of metaphyseal trabecular bone and cortical bone in the distal femur. (**d**,**e**) Quantitative micro-CT analyses of cortical and trabecular structure at the distal femur. Measurements were trabecular number (Tb.N), trabecular thickness (Tb.Th), trabecular (Tb) tissue volume (TV), bone volume (BV) and bone volume fraction (BV/TV), trabecular separation (Tb.Sp), bone mineral density (Tb.BMD), cotircal bone mineral density (Ct.BMD) and Cortical thickness (Ct.Th) (n = 3), WT or KO mice at 4 weeks old. Measurements are presented as mean ± SD. *p < 0.05 versus respective control.

Given that micro-CT could reveal microstructural changes such as trabecular and cortical bone morphology in the femur in which bone forms via cartilage ossification, we then evaluated the effect of PKD1 deficiency on bone mass and morphology by micro-CT, As shown in Fig. 3a, Representative images of the micro-CT analyses in the distal femur and epiphysis showed reduced bone mass, altered cortical thickness and structure of metaphyseal trabecular bone were observed in KO mice compared with WT groups. The transverse sectional images further displayed a moderately less trabecula in the proximal and the distal sites, and modestly thinner cortical layers in the KO groups (Fig. 3b). Moreover, the three dimensional images showed reconstruction of trabecular bones and cortical bones extending horizontally from the growth plate level to the distal diaphysis. This reconstruction region in KO mouse tissue revealed a sparse trabecular mass. In contrast, a more robust trabecular structure was found in WT control tissue (Fig. 3c).

When compared with littermate controls at the same ages, Osx::PKD1^fl/fl^ mice showed a significantly decrease in the trabecular thickness (Tb.Th), tissue volume (TV), bone volume (BV), bone volume fraction (BV/TV), bone mineral density (Tb.BMD), cortical thickness (Ct.Th) and cortical bone mineral density (Ct.BMD). There was a slight decrease in trabecular number (Tb.N) in 4-week-old Osx::PKD1^fl/fl^ mice. In contrast, the trabecular separation (Tb.Sp) was increased in Osx::PKD1^fl/fl^ mice compared with control mice (Fig. 3d,e). These data suggested an decreased bone mass phenotype in Osx::PKD1^fl/fl^ mice (KO).

### *In vivo* bone-specific conditional knockout of PKD1 affected osteoblastic development

**Figure 4:**
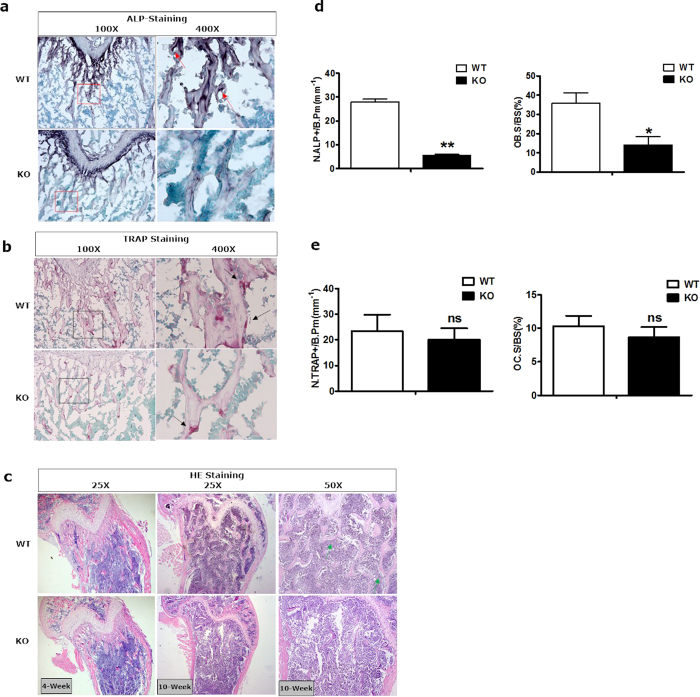
Bone-specific conditional knockout of PKD1 impaired osteoblasts development in the distal femur. (**a**) The activity of ALP was stained with purple in the membrane of osteoblast, as showed by red arrows. (**b**) The activity of TRAP was stained with red in osteoclasts, as showed by black arrows. (**c**) H&E staining of distal femur from 4 and 10 weeks KO and littermate control mouse. The green arrow indicated the trabecular bone. (**d**) Number of ALP positive cells/per bone perimeter (mm^−1^) (N.ALP+/B.Pm), osteolast surface per bone surface (OB.S/BS%). Measurements are presented as mean ± SD. *p < 0.05 versus respective control by t test. (**e**) Histomorphometric analysis showing no difference in osteoclast number per bone perimeter (mm^−1^) (N.TRAP+/B.Pm), and normal osteoclasts surface per bone surface (OC.S/BS,%) of WT and KO mice.

To assess the effect of PKD1 loss on the bone development, the activities of Alkaline Phosphatase (ALP) in the osteoblasts and of tartrate acid resistant phosphatase (TRAP) in the osteoclasts were analyzed by histological procedures. Compared with wild type mice, ALP positive osteoblastic lineage cells drastically decreased by 79% in trabecular bone from Osx::PKD1^fl/fl^ KO mice (Fig. 4a and d). Consistent with this, TRAP-positive multinucleated cells slightly reduced (approximately 14%) on the surface of trabecular bone at 4 weeks (Fig. 4b and e). Meanwhile, the decrease of trabecular number sustained from 4-week to 10-week PKD1-KO mice. Moreover, the decrease of trabecular bone was more severe in 10-week Osx::PKD1^fl/fl^ KO mice against 4-week Osx::PKD1^fl/fl^ KO mice (Fig. 4c). On the contrary, no significant difference was shown in cartilage growth plates and mineralized regions between KO mice and their littermate controls with HE and safranin O/fast green staining (see Supplementary Fig. 2S). These findings suggested that PKD1 deficiency in preosteoblasts may restrain the osteoblast differentiation and break the balance of osteoblasts and osteoclasts.

### PKD1 deficiency inhibited the expression of osteoblastic differentiation markers

**Figure 5:**
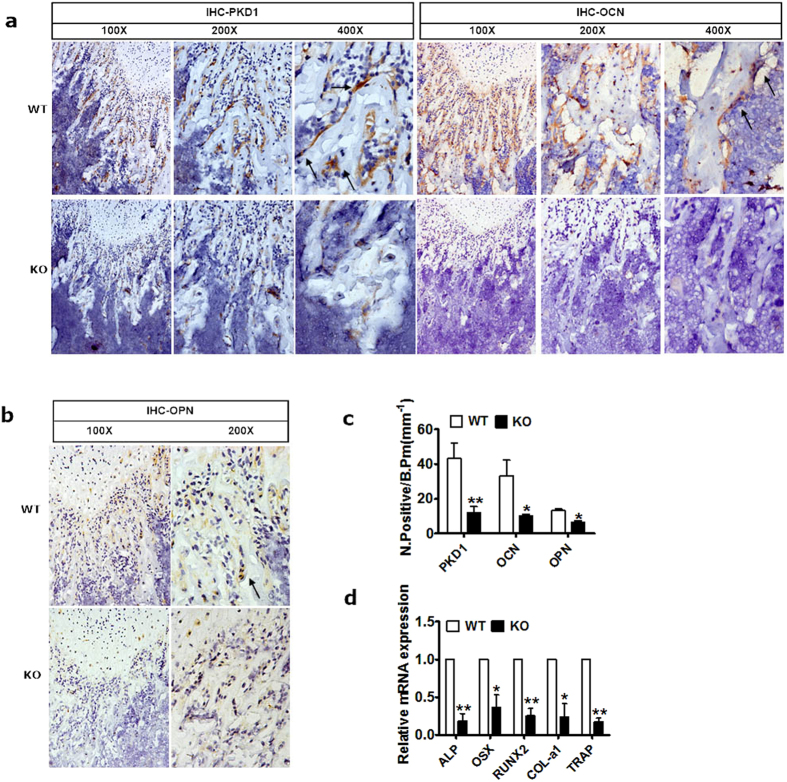
The expression of osteoblastic differentiation markers were inhibited by PKD1 deficiency. (**a**,**b**) IHC staining of PKD1 and osteoblastic markers OCN and OPN in femur from 4-week KO and control mouse. Positive osteoblasts were showed by black arrows. (**c**) Number of PKD1 positive, osteopontin positive (N.OPN^+^) and osteocalcin positive (N.OCN^+^) osteoblasts on the bone surface were measured as positive cells per millimeter of perimeter in sections (B.Pm). Measurements were presented as mean ± SD. *p < 0.05 versus respective control. The data in each group were analyzed using unpaired, two-tailed Student’s t-test. (**d**) Relative mRNA expression levels of osteoblast differentiation markers (ALP, OSX, Runx2, and col-α1) and osteoclast marker (TRAP) in long bone from wild-type and PKD1-KO female mice. The level of each marker gene expression was normalized to the level of the housekeeping gene. n > 3, WT and KO. *p < 0.05.

To determine whether the decreased bone mass caused by PKD1 deficiency was due to the inhibition of osteoblastic differentiation, we measured the expression of two osteoblastic differentiation markers: osteopontin (OPN) and osteocalcin (OCN)^22^. As shown in Fig. 5a,b, OPN and OCN expressions were remarkably inhibited in the Osx::PKD1^fl/fl^ KO mice, suggesting that restrained osteoblastic differentiation in the Osx::PKD1^fl/fl^ KO mice may arise from deficiency of PKD1. Moreover, The number of PKD1-positive osteoblasts, OPN-positive preosteoblasts and OCN-positive mature osteoblasts on the bone surfaces of Osx::PKD1^fl/fl^ KO mice were significantly reduced than that of WT mice (*p* < 0.05, Fig. 5c).

To further explore the effect of PKD1 deficiency on the specific molecular markers in the osteoblast lineage development, the mRNA levels in genes of interest in the long bone were measured. The forelimb bone from KO mice displayed a remarkable decrease in mRNA level of ALP, OSX, Runx2, collagenase I (COL-α1) (markers of osteoblast differentiation) and TRAP (marker of osteoclast differentiation) (*p* < 0.05, Fig. 5d). Taken together, deletion of PKD1 in mice impaired osteoblast development and decreased expression of osteoblastic regulators as well as osteoclasts differentiation.

### PKD1 was required for osteoblast development

**Figure 6:**
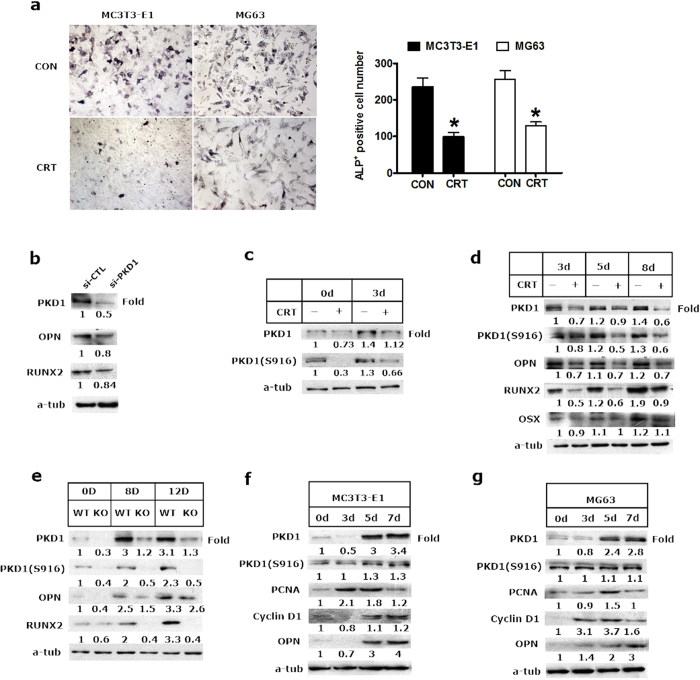
PKD1 contributed to osteoblastic development. (**a**) ALP staining and ALP-positive cell numbers of MC3T3-E1 and MG63 cells treated with 10 μM of CRT0066101(CRT) for 24 hours, positive numbers represent the mean ± SD of 3 wells (*p < 0.05). (**b**) MC3T3-E1 was transfected with siRNA of PKD1(si-PKD1) and negative control (si-CTL) for 48 hours. Osteoblastic markers (OPN and Runx2) and PKD1 expression were analyzed by Western blotting. The density of proteins bands was scanned and the values were normalized to control. (**c**) 0 day or 3 days differentiating MC3T3-E1 was treated with DMSO or 10 μM of CRT0066101 for 24 hours and then subjected to Western Blotting. (**d**) After days 3, 5, 8 differentiating, MC3T3-E1 cells were treated with CRT0066101 for 24 hours. Expression of PKD1 phosphorylation at ser916 and osteoblast regulators (OPN, Runx2 and OSX) were analyzed by Western Blotting. (**e**) Primary calvarial preosteoblast on the day of cell plating (day 0), on the 8^th^ and 12^th^ day of osteogenic differentiation were analyzed by Western blotting to detect expression of osteoblast markers Runx2, and OPN in WT or KO mice (n = 3). All Band densities were normalized to α-tublin content within each sample, and normalized to Day 0 at the far left WT group, control = 1.00. (**f**,**g**) PKD1, ser916 of PKD1phosphorylation, proliferative markers PCNA and Cyclin D1 were analyzed at the 0, 3^rd^, 5^th^ and 7^th^ day growth in the MC3T3-E1 and MG63 cells. Representative Western Blots were shown. Uncropped western blot images corresponding to Fig. 6(b,c,d). Figure 6(e,f and g) were shown in supplementary information file.

The family of PKDs is implicated in signal transduction of a wide range of biological responses, including changes of cell morphology, proliferation and differentiation^23^. To examine the cellular mechanisms underlying PKD1 regulation of osteoblasts development, we first investigated the effect of PKD inhibition on the activity of ALP in the murine preosteoblast MC3T3-E1cell line and MG63 osteoblast-like cells by CRT0066101, a PKD specific inhibitor^24^. Treatment with CRT0066101 remarkably reduced the ALP-positive cell number in MC3T3-E1 and MG63 cells (Fig. 6a) as well as ALP activity of MC3T3-E1 cells (Supplementary Fig. 3S). We then transfected with siRNA of PKD1 into MC3T3-E1 cells, Western blotting showed that Runx2 and OPN expression were also diminished by siRNA knockdown in MC3T3-E1 cells (Fig. 6b). These results suggested that activation of PKD1 was involved in the osteoblast differentiation *in vitro*.

Furthermore, we analyzed the effect of PKD1 on the expression of osteoblast differentiation markers during different days of osteogenic induction in MC3T3-E1cells. As shown in Fig. 6c, increased expression of PKD1 and its phosphorylation at ser916 by osteogenic induction in 3 days of MC3T3-E1 cells were diminished with CRT0066101 treatment, implying that PKD1 and its phosphorylation were upregulated at the early stage of osteoblast differentiation. Interestingly, the expression of osteoblast differentiation markers, Runx2, OPN and Osx, were also correlated with PKD1 and its phosphorylation at different day of osteoblast differentiation with or without CRT00661 treatment in MC3T3-E1 cells (Fig. 6d). Similarly, this temporal pattern of PKD1 expression, ser916 of phosphorylation, Runx2, OPN and Osx was observed in primary calvarial preosteoblasts subjected to differentiate for 12 days from PKD1-KO mice (Fig. 6e).

In addition, we investigated that the correlation of expression of PCNA and Cyclin D1 with PKD1 during different phage of proliferation. Western blotting showed that during the initial phase proliferation (day 0–5), PCNA and Cyclin D1 expression were gradually increased. By the 7^th^ day, PKD1 and OPN expression were markedly increased at this late stage of proliferation, but PCNA and Cyclin D1 expression were slightly decreased (Fig. 6f and g). Also, no significant cytotoxicity was observed in MC3T3-E1 and MG63 cells at 10 or 24 hours of treatment with 5 or 10 μM CRT0066101 compared with DMSO control (Supplementary Fig. 4S).

### PKD1 regulated osteoblast differentiation through STAT3 and p38 MAPK pathways

**Figure 7:**
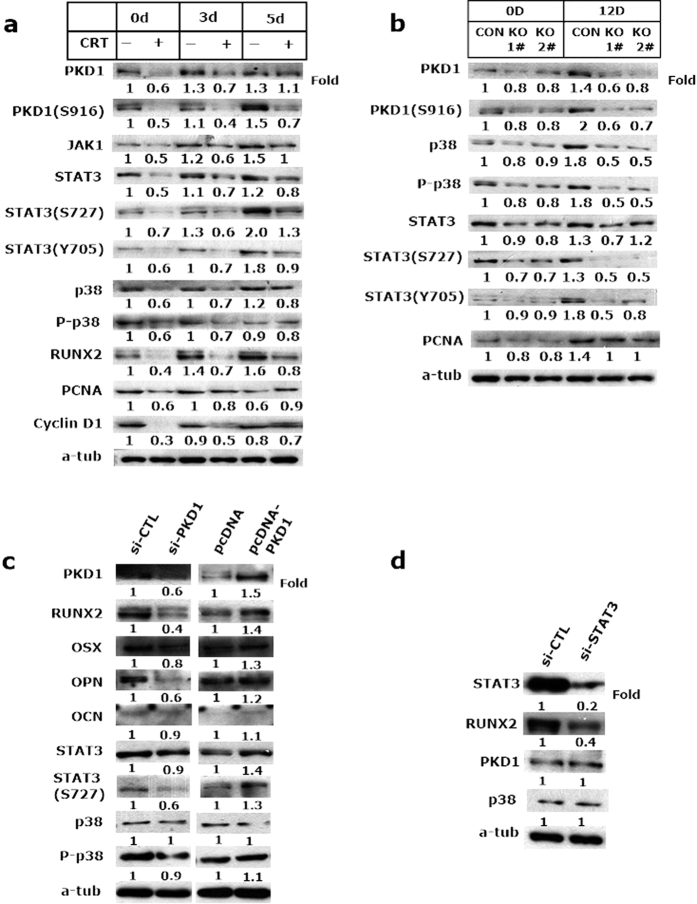
PKD1 impaired osteoblast differentiation through JAK1/STAT3 and p38 signaling pathway. (**a**) After days 3, 5 differentiating, MC3T3-E1 cells were treated with CRT0066101 or vehicle for 24 hours and then underwent immunoblotting to detect PKD1, PKD1 phosphorylation activity, JAK1, STAT3 and phosphorylation (ser727). Photoshop analyzed the density of bands on western blot. For each marker band, values were normalized to control and the density of control was set at 1. (**b**) Western Blot analysis of differentiating primary calvarial preosteoblasts showed decreased expression of p38,P-p38,STAT3,P-STAT3-ser727, y705 and PCNA in KO cells (0, 12^th^ day). n = 3, WT or KO mouse. (**c**) MC3T3-EI cells were transfected with si-PKD1 and negative control (si-CTL), pcDNA PKD1 and empty vector plasmid for 60 hours to analyze osteoblast regulators (Runx2, OPN,OSX and OCN) and relative pathway markers (STAT3 and p38). (**d**) MC3T3-EI cells were transfected with si-STAT3 and negative control (si-CTL) for 60 hours. Uncropped western blot images corresponding to Fig. 7(a,b,c and d) were shown in Supplementary Information file.

The signaling pathway induced by BMP-2 involves PKD activation of stress MAP-kinases JNK and p38, which play an important physiological role in osteoblastic cells^14^. It has been shown that a combined activation of PKC and STAT3 is essential for osteocytic-like differentiation^25^. To explore the effect of PKD on these signaling pathways, we first measured the expression and phosphorylation of JAK1, STAT3 and p38 in differentiating MC3T3-E1 cells after CRT0066101 treatment, As shown in Fig. 7a and Supplementary Fig. 5S, the expression and phosphorylation of JAK1, STAT3 and p38 were inhibited, while expression of PCNA and Cyclin D1 were progressively increased during osteoginc differentiation in PKD1 inhibitor treated group. Similarly, phosphorylation of STAT3 and p38 MAPK in PKD1-deficient calvarial cells derived from newborn Osx::PKD1^fl/fl^ mice was markedly decreased during osteoblast differentiation (Fig. 7b). These findings suggested that the STAT3 and p38 pathways may be potentially involved in osteoblastic differentiation. Moreover, silencing of endogenous PKD1 also reduced osteoblast regulators (Runx2, OSX, OPN and OCN) and phosphorylation of STAT3 and p38, whereas overexpression of PKD1 by transfected with PKD1 plasmid led to upregulate expression of osteoblastic markers and STAT3and p38 phosphorylation signals (Fig. 7c). In addition, siRNA-mediated knockdown of STAT3 resulted in a decrease of Runx2 activity without change of PKD1 or p38 expression (Fig. 7d). Taken together, our results demonstrated that PKD1 regulated the expression and phosphorylation of STAT3 and p38 signaling pathways, which may be needed for osteoblast differentiation *in vitro* and *in vivo*.

## Discussion

Using the Osx-Cre driver to conditionally inactivate PKD1 in preosteoblasts and block PKD1 secretion in these cells we found a severe low-bone-mass phenotype associated with decreased trabecular bone and inhibited osteoblast differentiation in this study. The loss of PKD1 was directly related to the reduction of osteoblastic differentiation activity *in vivo* and *in vitro*. Using conditional knockout mice and osteoblastic cell lines, we further defined the function of PKD1 involved in osteoblast development through the JAK1and STAT3 and p38 signaling pathways.

By establishing an Osx-controlled conditional PKD1 deletion mouse model, we found that weight and length of long bone were decreased as a result of PKD1 deficiency while organ/body weight ratios such as heart, lung, kidney and spleen were unchanged (see Supplementary Fig. 1S), which indicated that the overall weight loss of conditional KO mice may be primarily resulted from bone abnormalities. From 4-week to 10- week, the gap of whole body weight and limb bone length between WT and KO mice was sustained. In the early stages of postnatal growth, abnormal bone nodule in the scapula and less developed cranial cap were observed from 14 days of KO mouse bone staining by a loose mineralized bone structure along with delayed suture closure and calvarial defects. The reduced trabecular number, trabecular bone volume and cortical thickness from micro-CT analysis *in vivo* showed that decreased bone mass in PKD1 knockout mice, which resulted in postnatal growth bone defects *in vivo.*

Our study verified for the first time that conditional knockout PKD1 contributed to the decrease of bone mass characterized by declined trabecular bone. The result was consistent with that of Jeffery J^18^, which demonstrated that bone mineral density of the whole body and femoral bone compartments in heterozygous PKD1 (−/+) mice (PKD1 allele knock-in mice) were decreased compared with their wild-type littermates. However, the body weight, nasal-anal length, and percentage body fat of the mice were not significantly different from their wild-type littermates in their study, which differed from our findings. This phenotypic discrepancy was probably due to the PKD1 null genotype and bone-specific knockout in our study. Furthermore, it has been shown that PKD is important for growth plate bony repair and its inhibition after growth plate injury may result in less bone development and potentially more cartilage repair^26^. Taken together, based on our studies and other research, it is clear that PKD1 plays an important role in bone development and healing, and PKD1 may be involved in a positive feedback loop that promotes osteoblast differentiation. PKD1-targeted therapeutics may be effective for the treatment of bone abnormalities.

It was interesting to note that significant accumulation of total PKD1 activity was showed during the late stage of proliferation. During the initial phase of developmental (days 1–9 of culture), MC3T3-El cells were actively proliferating in cell number. By the 9^th^ day, cell cultures attained confluence, and underwent growth arrest, which was associated with beginning phase of osteoblast phenotypic development such as production of osteoblast differentiation marker of OPN^27^,^28^. During the initial phase proliferation (day 0–5), the expression of PCNA and Cyclin D1 gradually increased. The expression of the two proteins reached maximal around day 5. The changes of PKD1 expression did not correlated to the expression pattern of PCNA and Cyclin D1 during the period. In contrast, however, significant accumulation of total PKD1 activity showed in the late stage of proliferation (day 7), while PCNA and Cyclin D1 expression was markedly inhibited and cell cultures underwent growth arrest. During this period, an increased production of osteoblast differentiation marker OPN, characterized as the beginning of phenotypic osteoblast differentiation, was observed, which suggested PKD1 may associate with beginning of phenotypic osteoblast differentiation and be required for osteoblast differentiation (Fig. 6f,g).

It remains an open question whether defects of osteoblast development could alter homeostasis between bone development (osteoblast) and bone resorption (osteoclast)^4^. ALP and TRAP staining in our study also revealed declined ALP positive osteoblast cells in trabecular with statistical difference, while decreased TRAP positive multinucleated osteoclast in trabecular without statistical difference (Fig. 4d,e). These results showed that PKD1 knockout may lead to disruption of homeostasis of osteoblast and osteoclast: decreased expression of both. However, the decline of bone trabecular may not relate to multinucleated osteoclast formation. In limb bone of Osx::PKD1^fl/fl^ mice, qRT-PCR analysis of mature osteoblast marker ALP and osteoclast functional markers TRAP showed to be decreased. Interestingly, previous studies showed that based on qRT-PCR analysis of bone marrow macrophages treated with macrophage colony stimulating factor and RANKL (osteoclast differentiation regulator), PKD2 in most cases was highly expressed, with lower PKD3 level and even lower PKD1 levels in osteoclast, suggesting PKD2 may positively respond to stimuli of osteoclast differentiation^29^. PKD2 is most highly expressed in osteoclast precursors and during osteoclast differentiation, suggesting that PKD2 may play a unique role in osteoclastogenesis while PKD1 is the main isoform expressed in osteoblastic cells^30^. Although we could not rule out that the systemic effect of PKD1 acted on other osteocytes affecting bone mass indirectly, the results of PKD1 knockout in *vivo* were consistent with those from PKD inhibitor treatment *in vitro*, which re-affirmed our conclusion that PKD1 was essential for differentiation of preosteoblasts. It remains to be further investigated whether a decrease of PKD1 could result in decline of osteoclast cell numbers, or altered homeostasis between osteoblast and osteoclast.

Early research focused on the synergy of BMP-2 and PKD1 in osteoblast differentiation. For example BMP-2 and IGF-I signal through stimulated PKD involved in regulation of the expression of OSX and osteogenic differentiation^14^,^15^, BMP-2 could regulate osteoblast development by stimulating PKD1 via non-PKC-dependent pathways that activated JNK and p38 pathways^14^. Above studies have indicated that PKD1 regulates the development of the osteoblast through the BMP-2 signal. However, in the present study, we used specific inhibitors PKD1 CRT0066101 to investigate the direct effect of PKD1 on osteoblast lineage without stimulation of BMP-2. CRT0066101 could largely inhibit the activation of PKD1 measured by PKD1-ser916 phosphorylation, which resulted in inhibition of osteoblastic marker expression as compared with the control. The activity of PKD1 increased in days of osteoblast differentiation, and further supported the involvement of PKD1 in osteoblast development.

JNK and p38 are activated by BMP-2 and have been documented in the differentiating effect of this bone morphogenetic protein in MC3T3-E1 and primary calvarial-derived osteoblastic cells^14^. Recent studies indicated that BMP-2 can either activate the PI3K/Akt pathway in 2T3 cells^31^ or PKC pathways in human neonatal calvaria cells^32^,^33^. In MC3T3-E1 cells, ser 916 phosphorylation/activation of PKD has been implicated in the activation of JNK and p38 induced by BMP-2 to regulate osteoblast differentiation^14^. Studies have shown that the JAK/STAT and MAPK signaling pathways influence the development of osteoblast differentiation in MC3T3-E1 cells^34^, a mouse preosteoblast cell line. The JAK-STAT pathway is also linked to cell growth, proliferation, and differentiation in many cell types, including immune cells, hematopoietic stem cells, osteoblasts, neuronal precursor cells and myoblasts^29^,^35^,^36^. JAK1/STAT3 signaling depended on PKD1 expression in mediation of osteoblast differentiation (see Supplementary Fig. 4S). Complementary to these studies, our results showed for the first time that p38 and STAT3 activated by PKD1 may regulate osteoblast differentiation. Our data confirmed that activity of p38 and STAT3 decreased in calvaria cells from Osx-controlled conditional deletion of PKD1.

In summary, our study demonstrated a crucial role of PKD1 in bone development and osteoblast differentiation by establishing a novel bone-specific conditional PKD1-KO mouse model. Further analysis has identified STAT3 and p38 as critical downstream mediators of PKD1’s effects in osteoblast differentiation. Our findings using the PKD1 mutant mice have paved the way for systematic analysis of PKD in bone-related pathological conditionsand provided further support for developing novel PKD-targeted therapeutic conditions such as fracture healing and osteoporosis in a potentially cost-effective treatment.

## Materials and Methods

### Cell culture, siRNA transfection, and plasmid transfection

The preosteoblast cell line, MC3T3-E1, was cultured in alpha-MEM (Gibco) supplemented with 10% FBS (Gibco), 100 U/ml penicillin and 100 mg/mL streptomycin sulfate at 37 °C with 5% CO_2_, while the osteoblast-like cell line, MG63, was cultured in RPMI-164 (Hyclone). Primary osteoblastic cells were prepared from calvaria of wild type (WT) or knockout (KO) newborn mice as previously described^37^,^38^,^39^ and cultured using the same method as for MC3T3-E1 cells.

Small interfering RNAs (siRNAs) against PKD1 and STAT3 were designed to target the mRNA of mouse PKD1 (GenBankTM accession number NM_008858)^40^ and STAT3 (NC_000077.6) were transfected to MC3T3-E1 according to the manufacturer’s protocol (GenePharma). The plasmid pcDNA-PKD1 was transfected into cells by Hilymax (Dojindo) as suggested by the user manual.

### Proliferation and Differentiation Assay

For proliferation analysis, MC3T3-E1and MG63 cells were seeded in 12-well plates at a density of 5 × 10^4^ cells/well. The growth medium was changed every 2 days and cultured until confluent (7 days), which actively replicated and gradually increased in cell number. For osteogenic induction, 100 μg/ml ascorbic acid (Sigma-Aldrich) and 10 mM β-glycerol phosphate (Sigma-Aldrich) were added to confluent cells^41^. Cells without osteogenic induction treatment were referred to as the day 0 group. In day 0 group, cells were previously trypsinized and seeded overnight, then treated with 10 μM CRT0066101 for 24 hours. Then the cells were lysed for Western blotting. The cells under osteogenic induction were treated for 3, 5, 8 and 12 days, and the medium was then replaced with fresh medium containing 10 μM CRT0066101. For inhibitor studies the cells were treated with dimethyl sulfoxide (DMSO) or 10 μM CRT0066101 (Sigma-Aldrich, 956123-34-5) dissolved in DMSO for 24 hours.

### Animals and Genotyping

Newborn C57BL/6 mice were purchased from the Laboratory Animal Centre of Southern Medical University. PKD1^flox/flox^ mice (014181-B6) were purchased from the Jackson lab^19^, and Osx-GFP::Cre (006361-B6)^42^ were generously provided by Dr. Xiaochun Bai from Southern Medical University. This mouse owns the expression of a tetracycline (Tet)-Off regulatable GFP-Cre fusion protein that is transcriptionally controlled by an Osx promoter. These two strains were crossed and maintained on a C57BL/6J background. We first generated double heterozygous mice for Cre and floxed PKD1 (Osx::PKD1^fl/+^) that were then bred to PKD1^fl/fl^ mice via a back-mating strategy to generate excised floxed PKD1 homozygous (Osx::PKD1^fl/fl^; used as conditional knock-out), and Cre-null mice (PKD1^fl/+^ and PKD1^fl/fl^); used as littermate controls throughout the study. These mice were born at the expected Mendelian frequency, while high death rate in knock-outs mice because of defects in weight and bone development. Genotyping was conducted by PCR of tail lysate (Primers see the Supplementary Table 1).

Mice used in the study were housed in specific pathogen-free conditions. All animal protocols and experiments were approved by the Institutional Animal Care and Use Committees of Southern Medical University and were performed in accordance with the relevant guidelines.

### Micro-CT analysis

After 48-hour 4% paraformaldehyde fixing, female mice left femora was performed for quantitative analysis. In brief, microarchitecture of the distal trabecular bone and midshaft cortical bone of the femur were measured by ScancoμCT 80^43^. Bones were placed vertically in 12-mm-diameter scanning holders and scanned at the following settings: 12-μm resolution, 55-kVp energy, 145-μA intensity, and an integration time of 200 ms. Considering that groups had conditions that affected bone length, in fact we scaned almost 30% of total femur length from the growth plate and extended axially towards the diaphysis. In transverse slices of this region of interest (ROI), we started morphometric analysis from the proximal edge of the growth plate and extending towards the diaphysis for 60 slices, which was delineated manually matching with the area occupied by trabecular or cortical bone to perform trabecular or cortical bone analysis respectively. Using a contouring tool, we segmented the trabecular bone from the cortical shell manually on key slices, and morphed the contours automatically to segment the trabecular bone on all slices. The three-dimensional structure and morphometry were constructed and analyzed for BV/TV (%), Tb.N. (mm^−1^), Tb.Th. (mm) Tb.Sp (mm), Tb.BMD (mg/cm^−3^), Ct.Th and Ct.BMD (mg/cm^−3^)^44^,^45^.

### RT-PCR, Real Time PCR, and Western Blot

Total RNA of mouse tissues was extracted using the RNeasy extraction kit (Qiagen), and an equal amount of 1 μg of total RNA per sample was reverse transcribed. All-in-One First-Strand cDNA Synthesis Kit and All-in-One™ qPCR Mix were from GeneCopoeia. Primer sequences were listed in Supplementary Table 1. The level of target gene expression was normalized with the housekeeping gene GAPDH.

Cells and tissues were lysated by 2% sodium dodecyl sulfate with 2 M urea, 10% glycerol, 10 mM Tris-HCl (pH 6.8), 10 mM dithiothreitol and 1 mM phenylmethylsulfonyl fluoride. The lysates were centrifuged and the supernatants were separated by SDS-PAGE and blotted onto a nitrocellulose (NC) membrane (Bio-Rad Laboratories). The membrane was then analyzed using specific antibodies and visualized by enhanced chemiluminescence (ECL Kit, Amersham Biosciences). Proteins were detected by immunoblotting with antibodies against PKD1(CST, 1:1000,#2052), PKD1ser916(CST, 1:1000,#2051), Runx2(Abclonal, 1:500, A2851), OSX (Bioss, 1:1000, bs-1110R), OPN (Bioworlde, 1:1000, BS1264), cyclineD1(CST, 1:1000,#2922), PCNA (Abclonal, 1:1000, A4006), STAT3(bioworlde, 1:1000, BS1335), STAT3phospho-ser727(bioworlde, 1:500, BS4180), STAT3phospho-Y705 (bioworlde, 1:500, BS4181), p38MAPK (CST, 1:1000,#9212), P-p38 (CST, 1:1000,#4511P).

### ALP and TRAP Staining

To measure ALP activity, after twice washings in PBS for each 10 sec and 4% paraformaldehyde fixing for 15 minutes, cells were incubated for 1 hour at room temperature with an alkaline phosphatase staining. Detection was performed by the BCIP/NBT Alkaline Phosphatase Color Development Kit (Beyotime Institute of Biotechnology) according to the manufacturer’s instructions, which was counterstained by fast green. The EDTA-treated sections were stained for tartrate acid resistant phosphatase (TRAP) and counterstained with fast green. For histomorphometric analyses, ALP and TRAP-stained sections were used for quantification of osteoblast and osteoclast, number and surface, using Image Pro Plus 6.0 software (Media Cybernetics, MD, USA).

### Bone histology and immunohistochemistry Analysis

Femur tissues dissected from the mice were fixed using 4% paraformaldehyde in PBS at 4 °C for 24 hours and then decalcified in 15% EDTA (pH 7.4) at 4 °C for 14 days. The tissues were embedded in paraffin or optimal cutting temperature (OCT) compound (Sakura Finetek), and 2–5 μm sagittal-oriented sections were prepared for histological analyses. H&E staining was performed as previously described^46^. Tartrate-resistant acid phosphatase (TRAP) or alkaline phosphatase (ALP) staining was performed using a standard protocol (Sigma-Aldrich), which were counterstained by fast green. For IHC, we incubated primary antibodies which recognized mouse PKD1 (Elabscience, 1:80, ESAP12972), OPN (bioworlde, 1:50, BS1264), OCN (proteintech, 1:50, 23418-1-AP) overnight at 4 °C. All sections were observed and photographed on Olympus BX51 microscope. In immunohistochemical assays, cells per bone perimeter (B.Pm) was used to calculate the number of positive cells in 6 different images taken at 100x magnification with Image Pro Plus 6.0 software (Media Cybernetics, MD, USA)^47^. At least three mice per group were examined.

### Statistics

All results were presented as the mean ± SD. Calculated using SPSS20.0 software and GraphPad Prism 5.0 (Graphpad, San Diego, CA). Differences between two experimental groups were compared by the unpaired Student’s test. SPSS software was used for statistical analysis, and the results were considered significantly different at a value of *p* < 0.05.

## Additional Information

**How to cite this article**: Li, S. *et al*. A Conditional Knockout Mouse Model Reveals a Critical Role of PKD1 in Osteoblast Differentiation and Bone Development. *Sci. Rep.* **7**, 40505; doi: 10.1038/srep40505 (2017).

**Publisher's note:** Springer Nature remains neutral with regard to jurisdictional claims in published maps and institutional affiliations.

## Supplementary Material

Supplementary Information
