# Supplementary material for: A Conditional Knockout Mouse Model Reveals a Critical Role of PKD1 in Osteoblast Differentiation and Bone Development
Source: Sci Rep. 2017 Jan 13;7:40505. doi: 10.1038/srep40505 (PMC5233966; doi:10.1038/srep40505)
Supplement: Supplementary Information [file srep40505-s1.pdf]

A Conditional Knockout Mouse Model Reveals Critical Role of PKD1 in Osteoblast Differentiation and Bone Development

Shao Li[1], Wanfu Xu[1], Zhe Xing[1], Jiabi Qian[1], Liping Chen[1], Ruonan Gu[1], Wenjing Guo[1], Xiaoju Lai[1], Wanlu Zhao[1], Songyu Li[1], Yaodong Wang[1], Q. Jane Wang[2] and Fan Deng[1*]

[1]Department of Cell Biology, School of Basic Medical Sciences, Southern Medical University, Guangzhou 510515, P. R. China;

[2]Department of Pharmacology and Chemical Biology, University of Pittsburgh School of Medicine, Pittsburgh, PA 15261.

Supplementary Figures and Legends

Figure 1S. Weight alternation in OSX::PKD1$^{fl/fl}$ mice. A. the body weight of the 4-week-old and 10-week-old PKD1-deficient mice. B. Weight ratio of organ such as heart, lung, kidney, spleen and liver from 4-week-old WT and KO mouse. C. Body weight change between WT and PKD1-KO mice in 4-week, 10-week and 12-week. The data in each group were analyzed using unpaired, two-tailed Student's t-test. The level of significance was set at *, $p<0.05$.

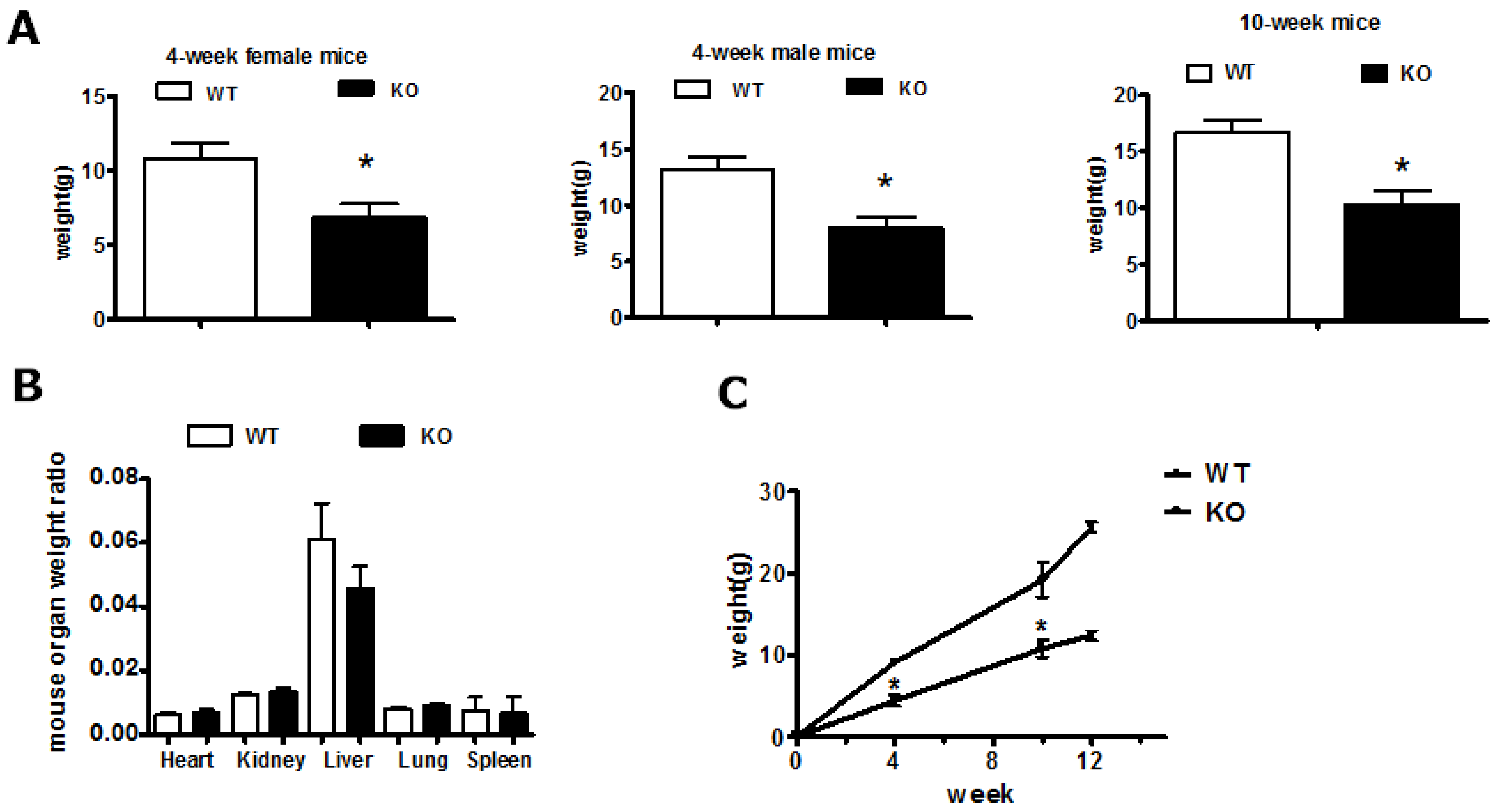
A
4-week female mice
WT
KO
weight(g)
15
10
5
0
*
4-week male mice
20
15
10
5
0
10-week mice
B
mouse organ weight ratio
0.08
0.06
0.04
0.02
0.00
Heart
Kidney
Liver
Lung
Spleen
C
WT
KO
30
20
10
0
4
8
12
week

Figure 2S. Growth plate size and cartilage matrix in WT and OSX::PKD1$^{fl/fl}$ mice A) H&E staining and safranin O/ fast green staining(the left panels) of the distal femur showing normal growth plate and cartilage matrix in in 4-week-old KO compared with WT mice. Magnification: 100x for the left and right panels,400x for the middle panels. (B) Growth plate measurements showing no statistical difference in total growth plate (total gp) and proliferative zone length (p), and normal hypertrophic zone length (h), in 4-week-old KO compared with WT mice. Data were normalized to the total growth plate length of WT mice (n=3).

A

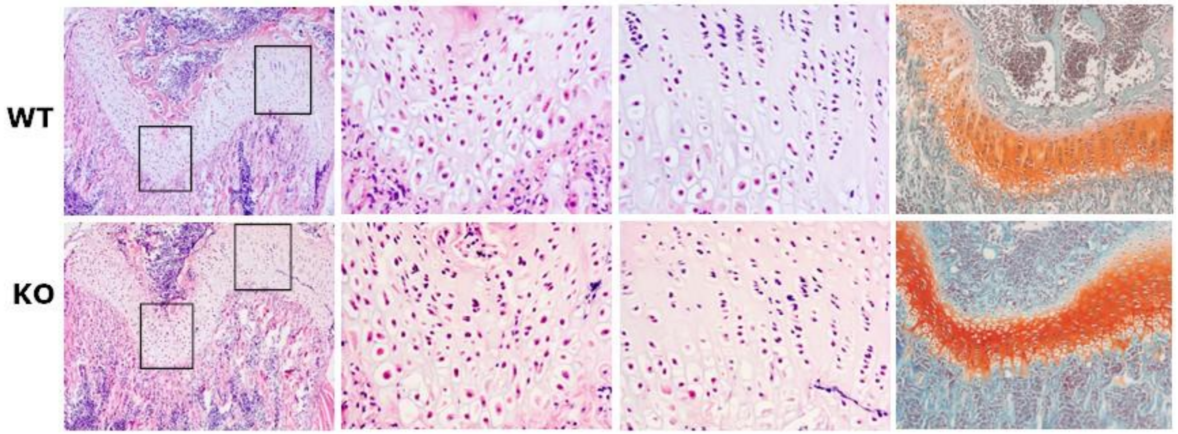

B

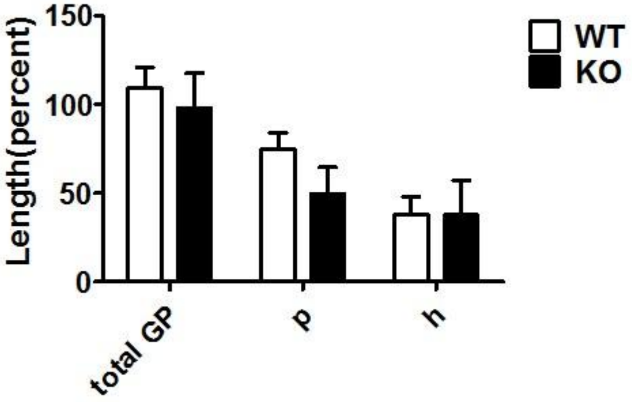

Figure 3S. ALP activities with CRT0066101 treatment during osteogenic differentiation in MC3T3-E1. ALP staining of MC3T3-E1 treated with DMSO (con) and CRT0066101 in 0th,3th,5th,7th differentiation day.

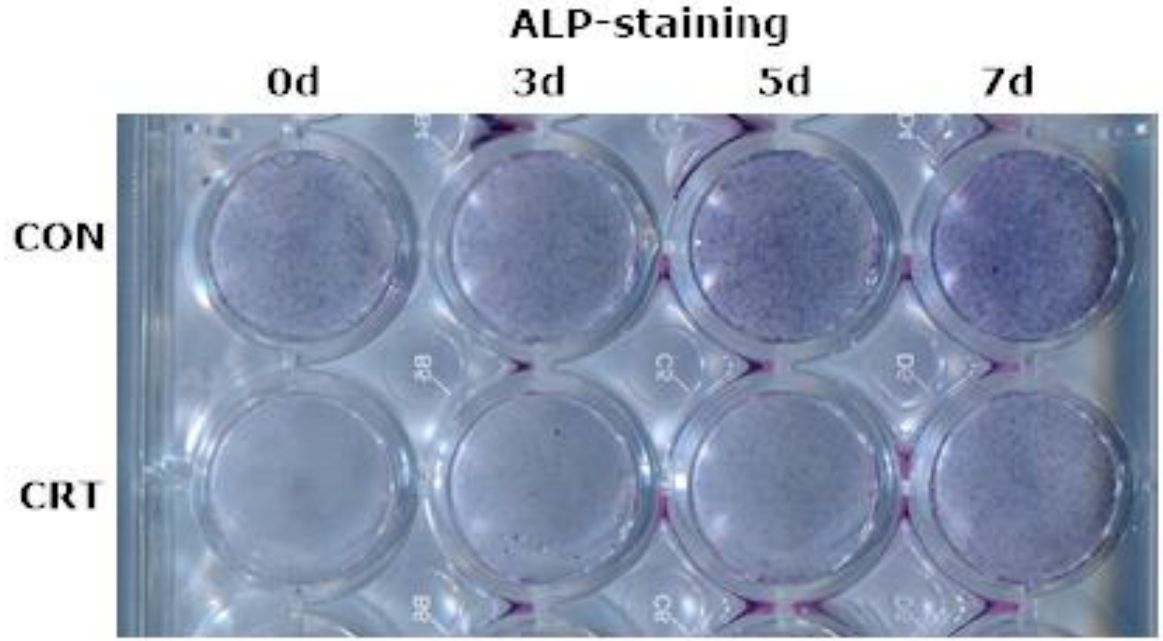

Figure 4S. **The cytotoxicity of PKD inhibitor CRT on MC3T3-E1 and MG63 cell lines by CCK-8 assay.** Cell viability for CRT in cells was measured for indicated time compared to DMSO control. Percent viability of cells was expressed relative to control cells (n=3, presented as the mean ±SD).

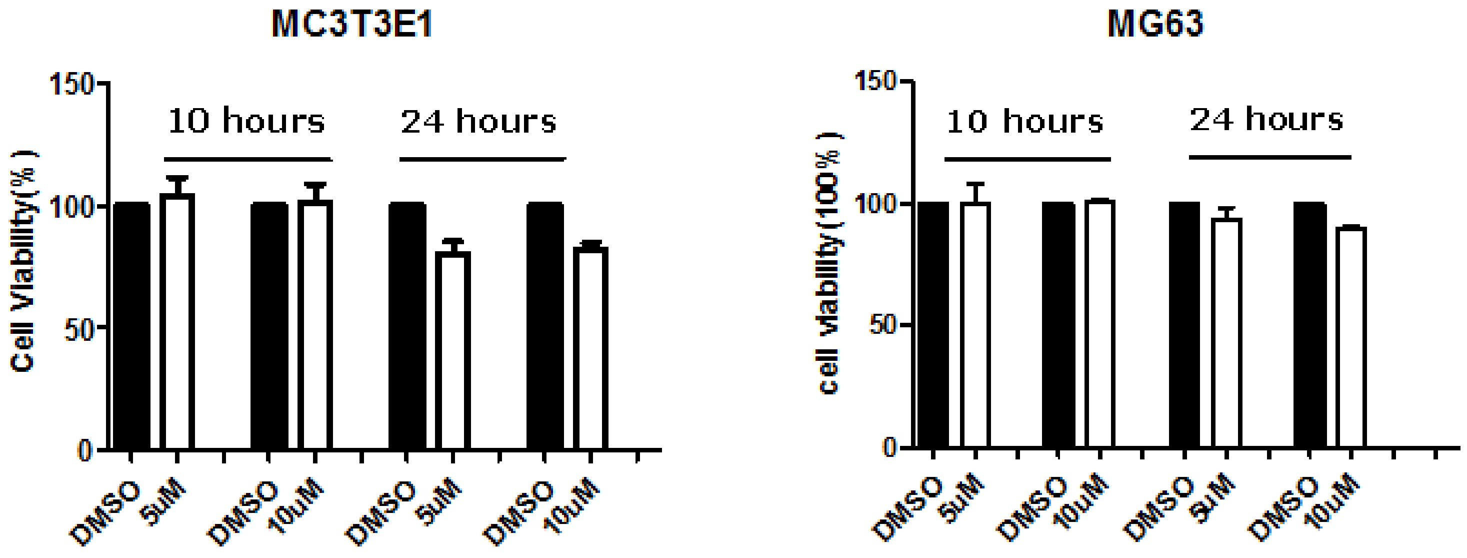

Figure 5S. The effect of PKD1 phosphorylation (ser916) on JAK1/STAT3 signaling in mediation of osteoblast differentiation. Confluent MC3T3-E1 cells were preincubated with PMA(100nM)or their vehicle for 3 hours and then exposed to CRT0066101(10μM) for 24 hours then collected protein was analyzed by Western blot to detect expression of JAK1,STAT3,STAT3-ser705, STAT3-ser727 and osteoblastic markers.

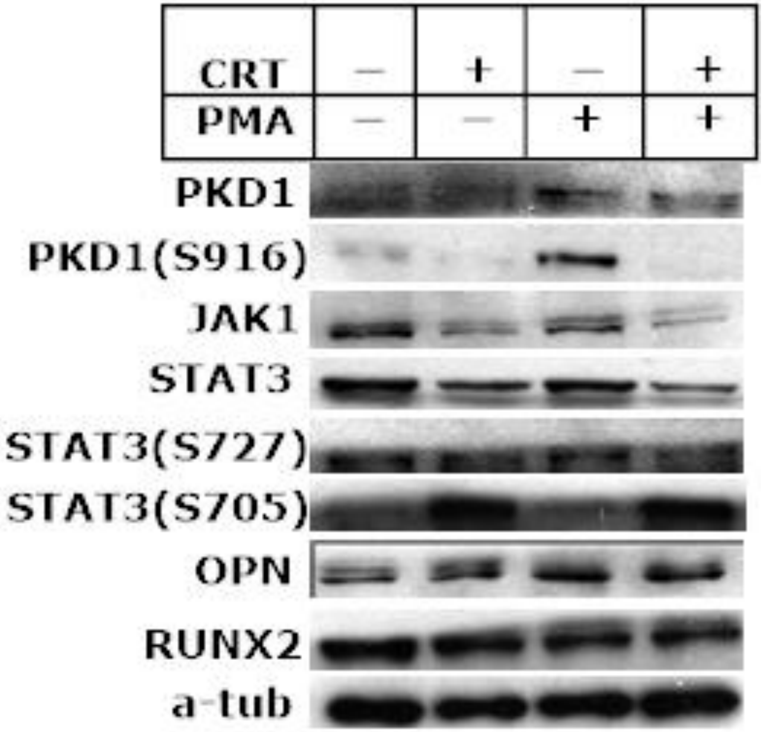

Supplementary Figure 1. PKD1 was deleted specifically in transgenic mice. Uncropped western blot images corresponding to Figure 1(b), Figure 1(c)

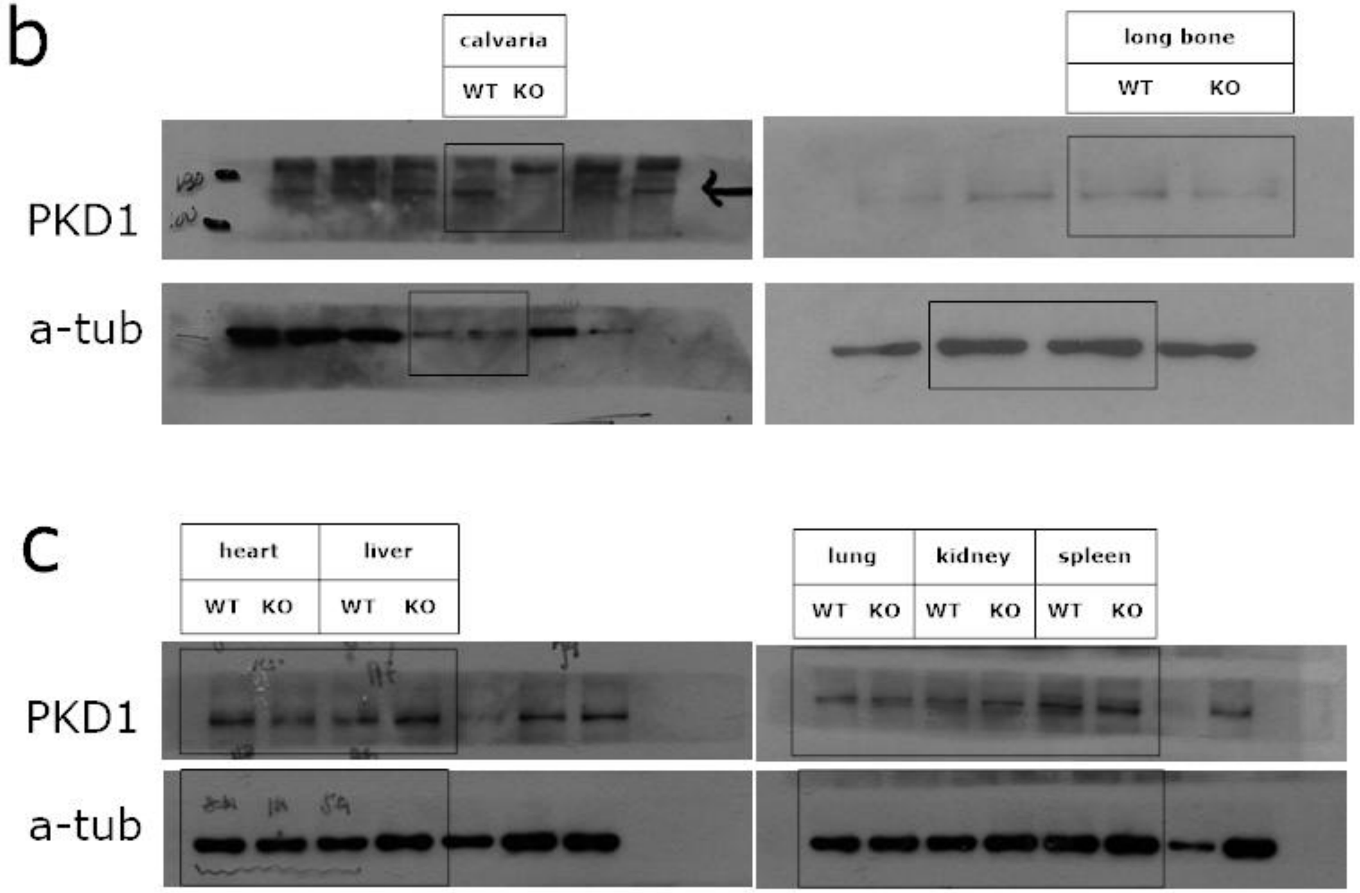
b
calvaria
WT KO
long bone
WT KO
PKD1
a-tub
c
heart
liver
WT KO WT KO
lung
kidney
spleen
WT KO WT KO WT KO
PKD1
a-tub

Supplementary Figure 6. PKD1 contributed to osteoblastic development. Uncropped western blot images corresponding to Figure 6(b), Figure 6(c) ,Figure 6(d). Figure 6(e), Figure 6(f) and Figure 6(g)

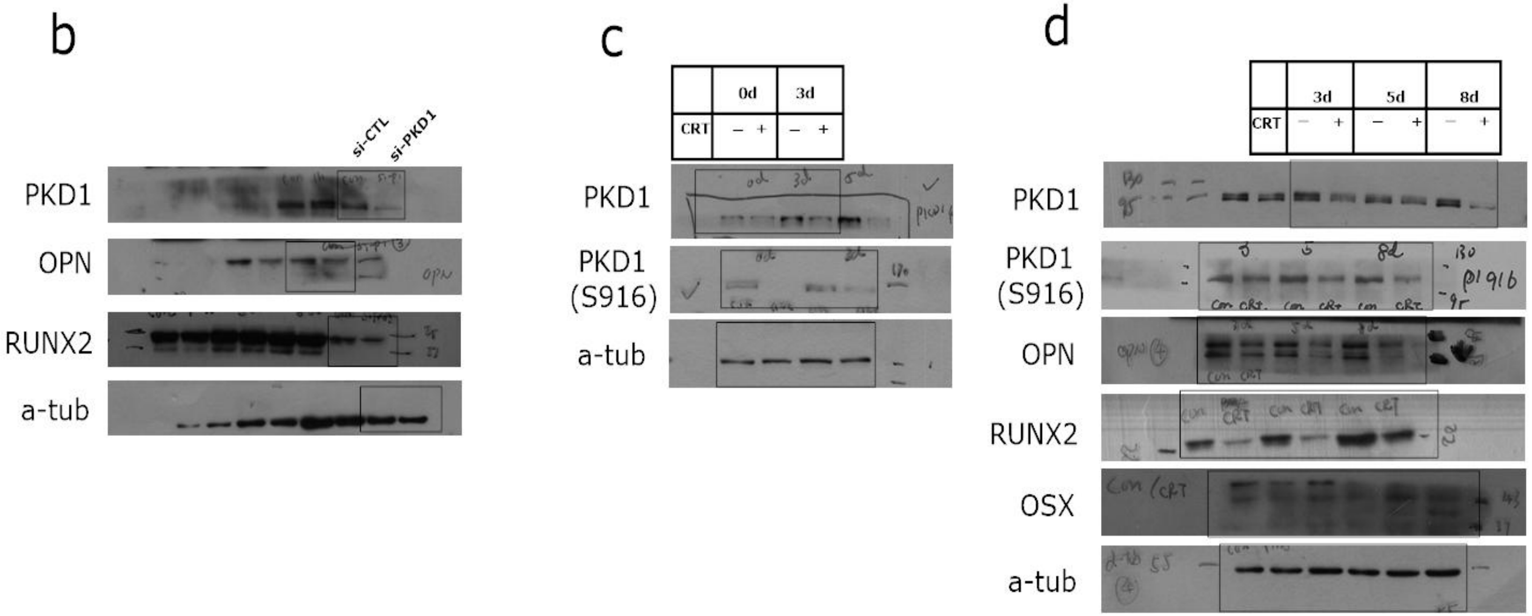

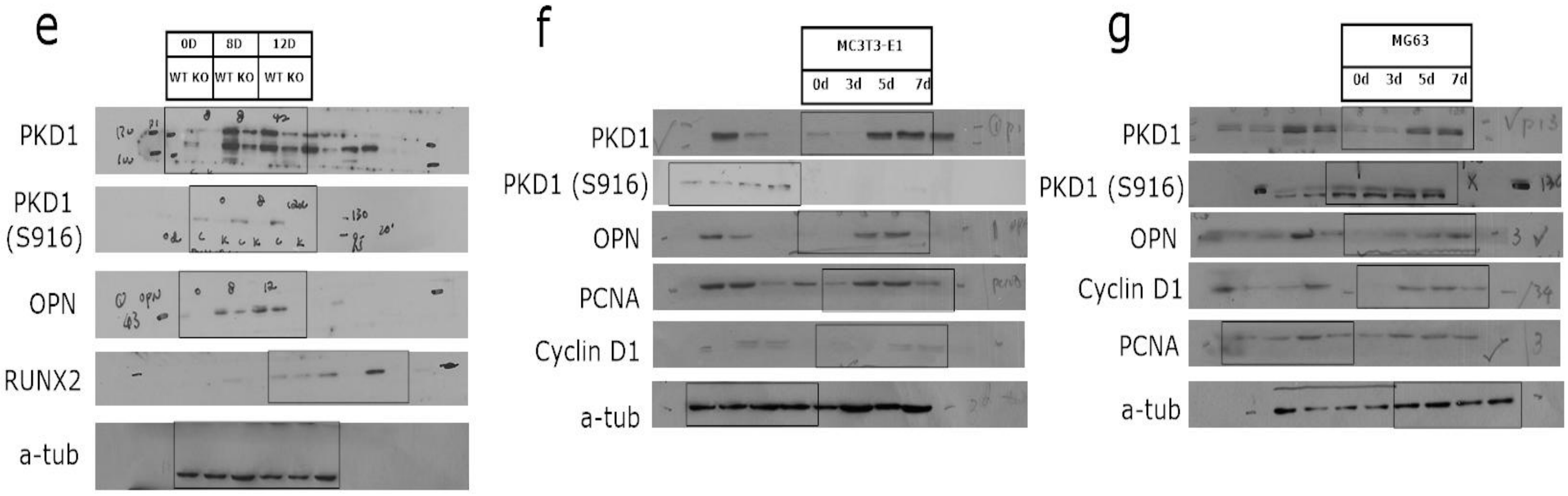

Supplementary Figure 7. PKD1 impaired osteoblast differentiation through JAK1/STAT3 and p38 signaling pathway.

Uncropped western blot images corresponding to Figure 7(a), Figure 7(b) ,Figure 7(c) and Figure 7(d).

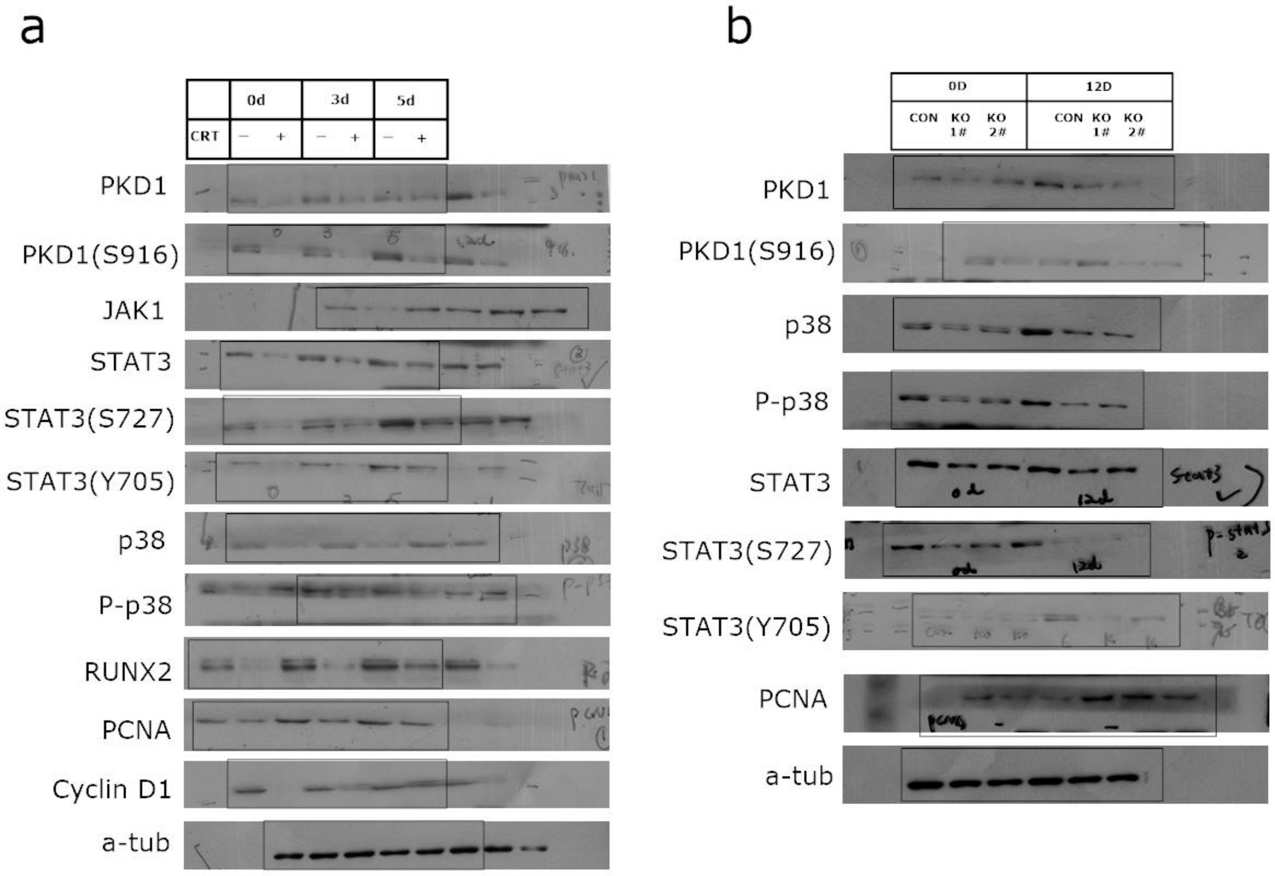
a
0d
3d
5d
CRT
− + − + − +
PKD1
PKD1(S916)
JAK1
STAT3
STAT3(S727)
STAT3(Y705)
p38
P-p38
RUNX2
PCNA
Cyclin D1
a-tub
b
0D
12D
CON KO 1# KO 2#
CON KO 1# KO 2#
PKD1
PKD1(S916)
p38
P-p38
STAT3
STAT3(S727)
STAT3(Y705)
PCNA
a-tub

c

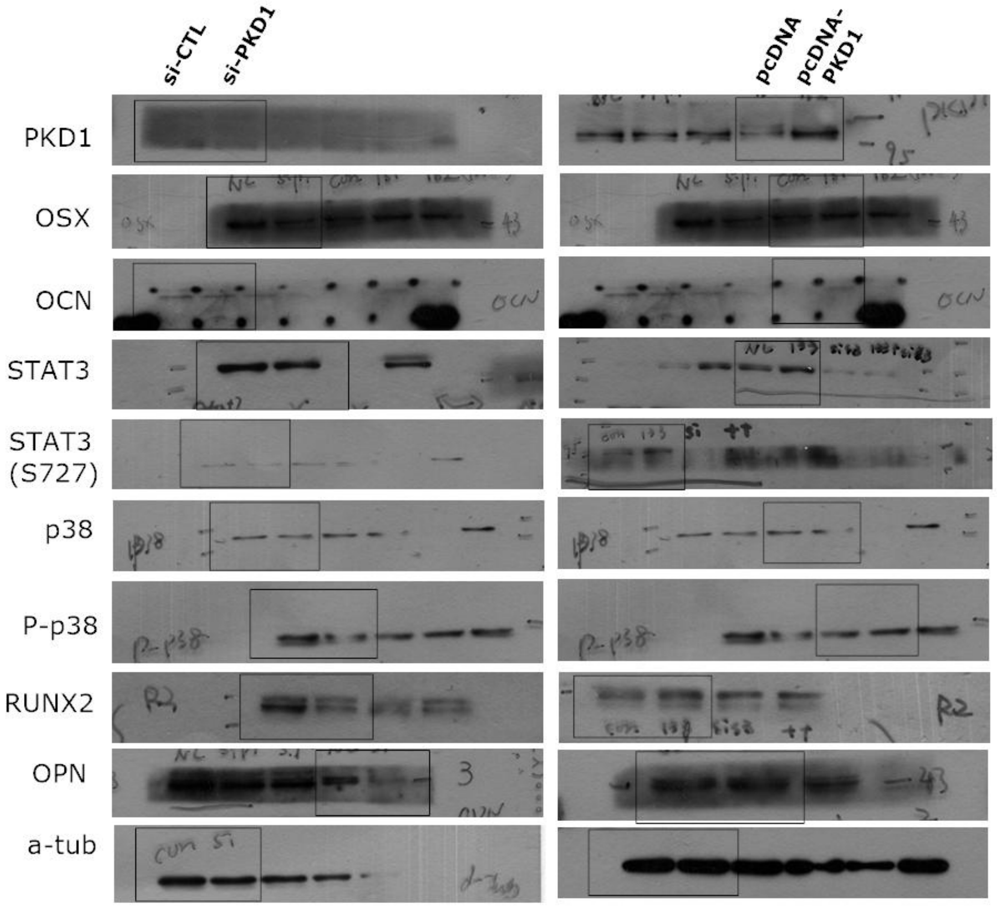
si-CTL
si-PKD1
pcDNA
pcDNA-PKD1
PKD1
OSX
OCN
STAT3
STAT3 (S727)
p38
P-p38
RUNX2
OPN
a-tub

d

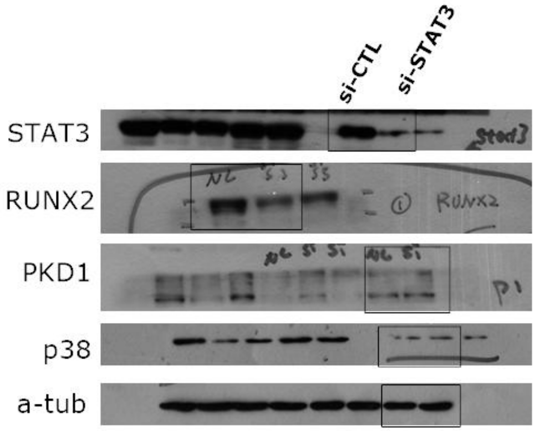
si-CTL
si-STAT3
STAT3
RUNX2
PKD1
p38
a-tub

Table 1. Real-time RT-PCR primers and genotyping primers for mice: primer sequences of osteoblast markers Col (I), ALP, OCN, RUNX2, TRAP and OSX; primer sequences for Cre transgene and PKD1 floxed gene. Primer sequences used in PCR and Real time RT-PCR.

**Target gene**	**Sequences(5'-3')**
Col(I)-Forward	GCG AAG GCA ACA GTC GCT
Col(I)-Reverse	CTT GGT GGT TTT GTA ATT CGA TGA C
ALP-Forward	TCC TGA CCA AAA ACC TCA AAG G
ALP-Reverse	TGC TTC ATG CAG AGC CTG C
OCN-Forward	CTC ACA GAT GCC AAG CCC A
OCN-Reverse	GCG CCG GAG TCT GTT CAC TA
RUNX2-Forward	GGC TCT GGC GTT TAA ATG GTT
RUNX2-Reverse	GTG CCC TCT GTT GTA AAT ACT GCT T
TRAP-Forward	GTA CCA GGG CAG AGA AGC TG

TRAP-Reverse	AAC ACC ACG AGA GTC CTG CT
OSX-Cre-0872	AAG TTC ATC TGC ACC ACC G
OsX-Cre-1416	TCC TTG AAG AAG ATG GTG CG
PKD1-Floxed-F	GCC CAC AGC TAT TGT TCC TAA
PKD1-Floxed-R	GGA TAA AGT GAT CAA GCA GCA
